# Supplementary figures and images for: The Adipokinetic Hormone (AKH) and the Adipokinetic Hormone/Corazonin-Related Peptide (ACP) Signalling Systems of the Yellow Fever Mosquito Aedes aegypti: Chemical Models of Binding
Source: Biomolecules. 2024 Mar 6;14(3):313. doi: 10.3390/biom14030313 (PMC10968007; doi:10.3390/biom14030313)

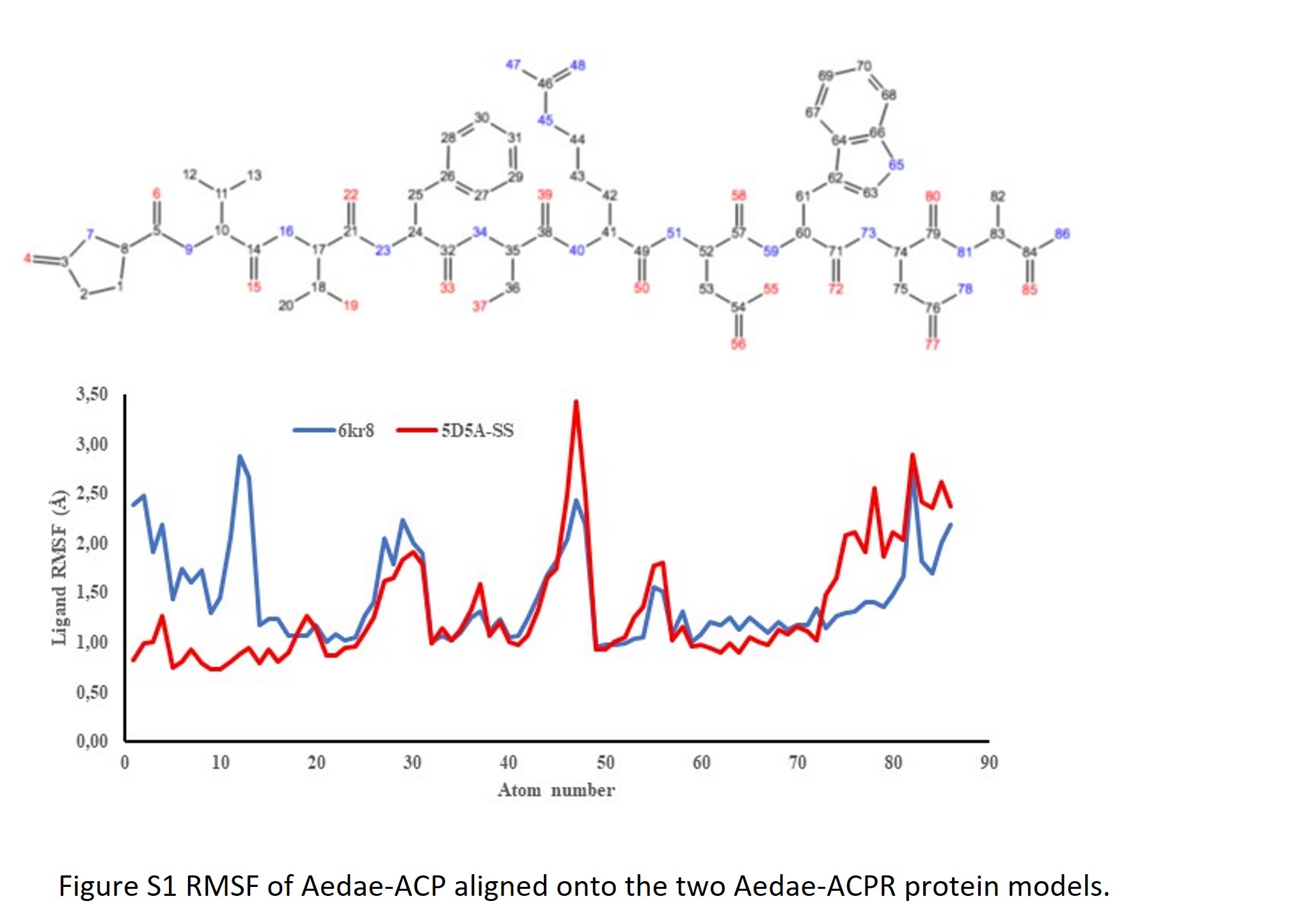

Supplement: Supplementary file 1 [file biomolecules-14-00313-s001.zip › biomolecules-2849093-supplementary/Figure S1.jpg]

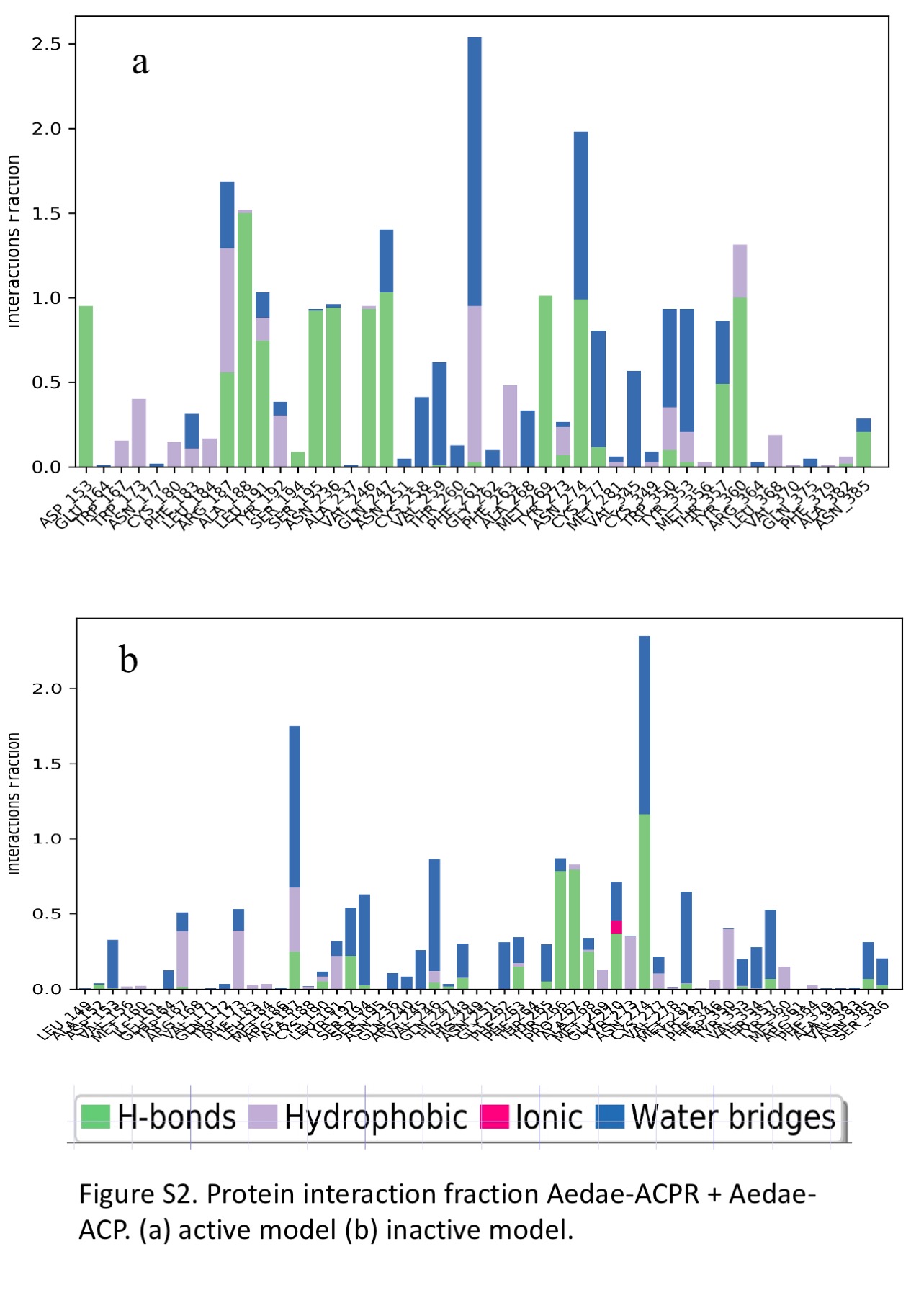

Supplement: Supplementary file 1 [file biomolecules-14-00313-s001.zip › biomolecules-2849093-supplementary/Figure S2.jpg]

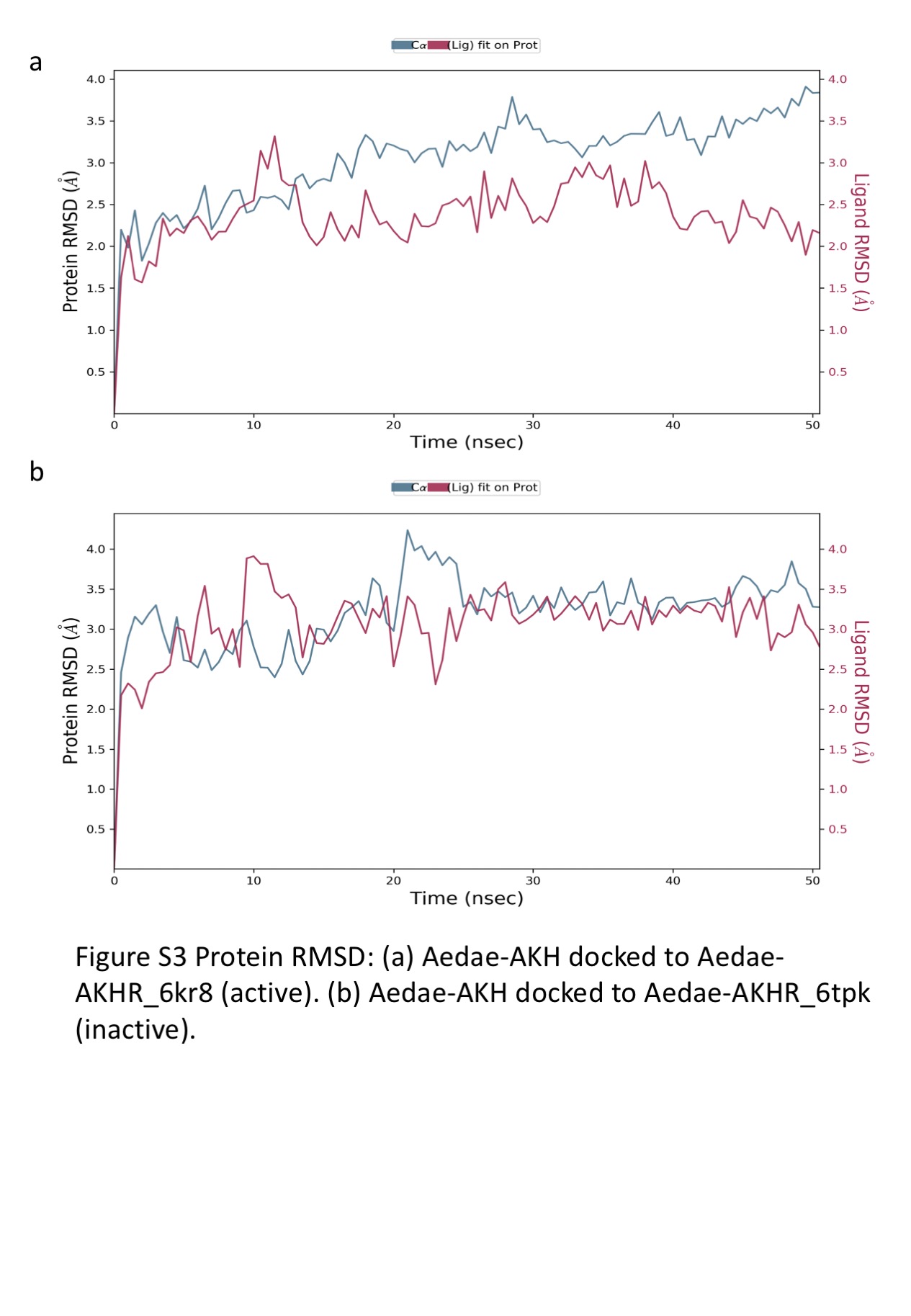

Supplement: Supplementary file 1 [file biomolecules-14-00313-s001.zip › biomolecules-2849093-supplementary/Figure S3.jpg]

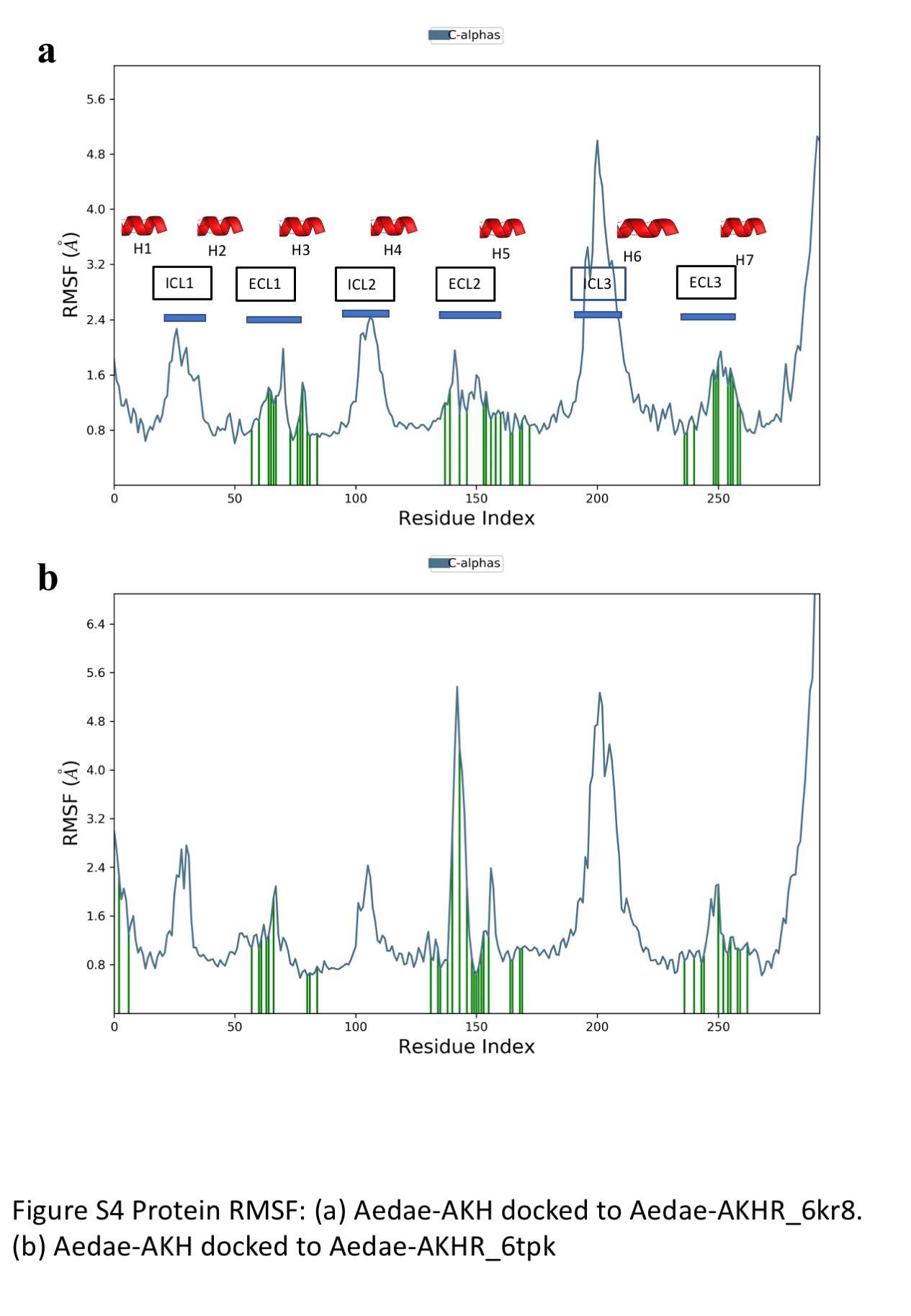

Supplement: Supplementary file 1 [file biomolecules-14-00313-s001.zip › biomolecules-2849093-supplementary/Figure S4.jpg]

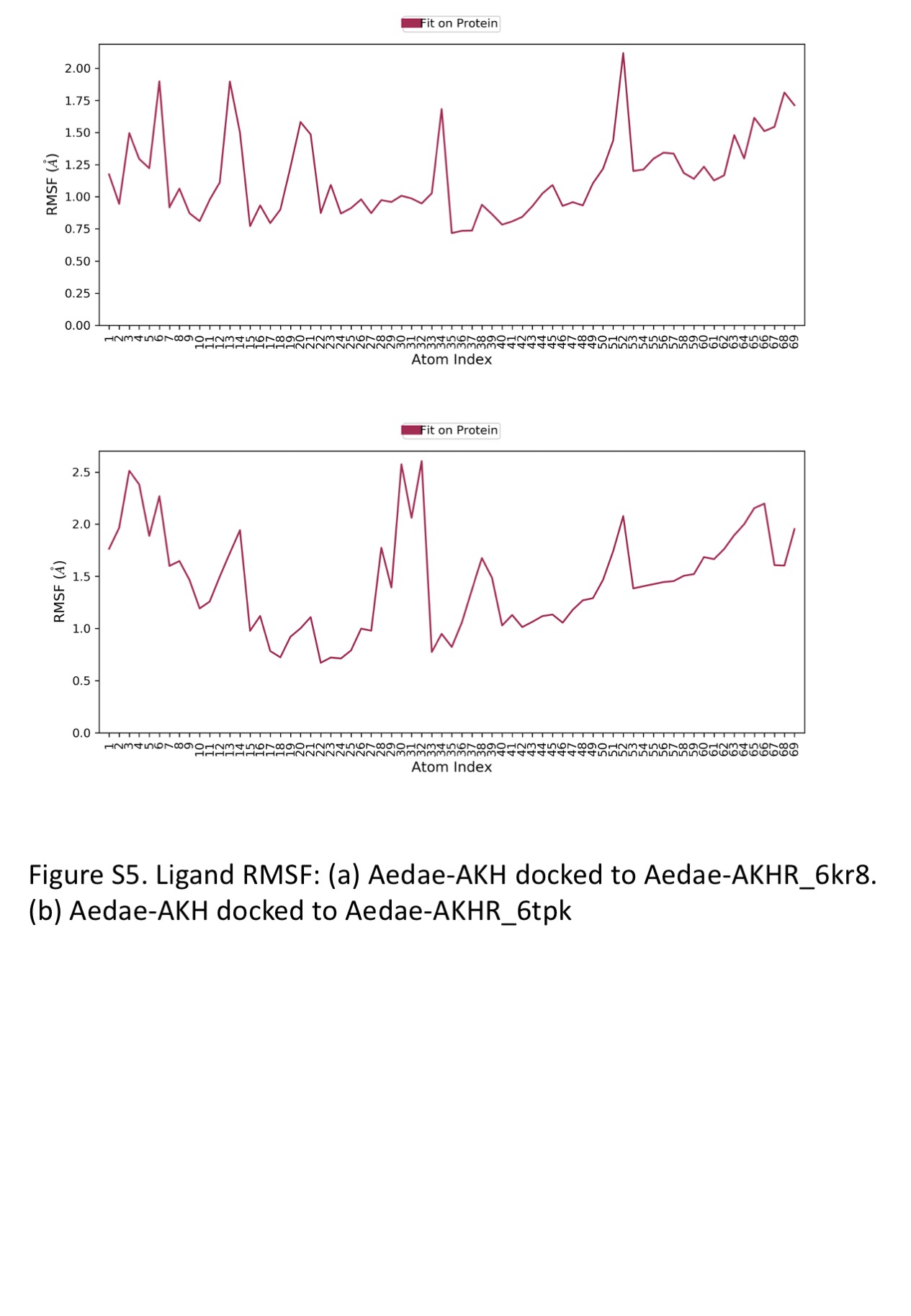

Supplement: Supplementary file 1 [file biomolecules-14-00313-s001.zip › biomolecules-2849093-supplementary/Figure S5.jpg]

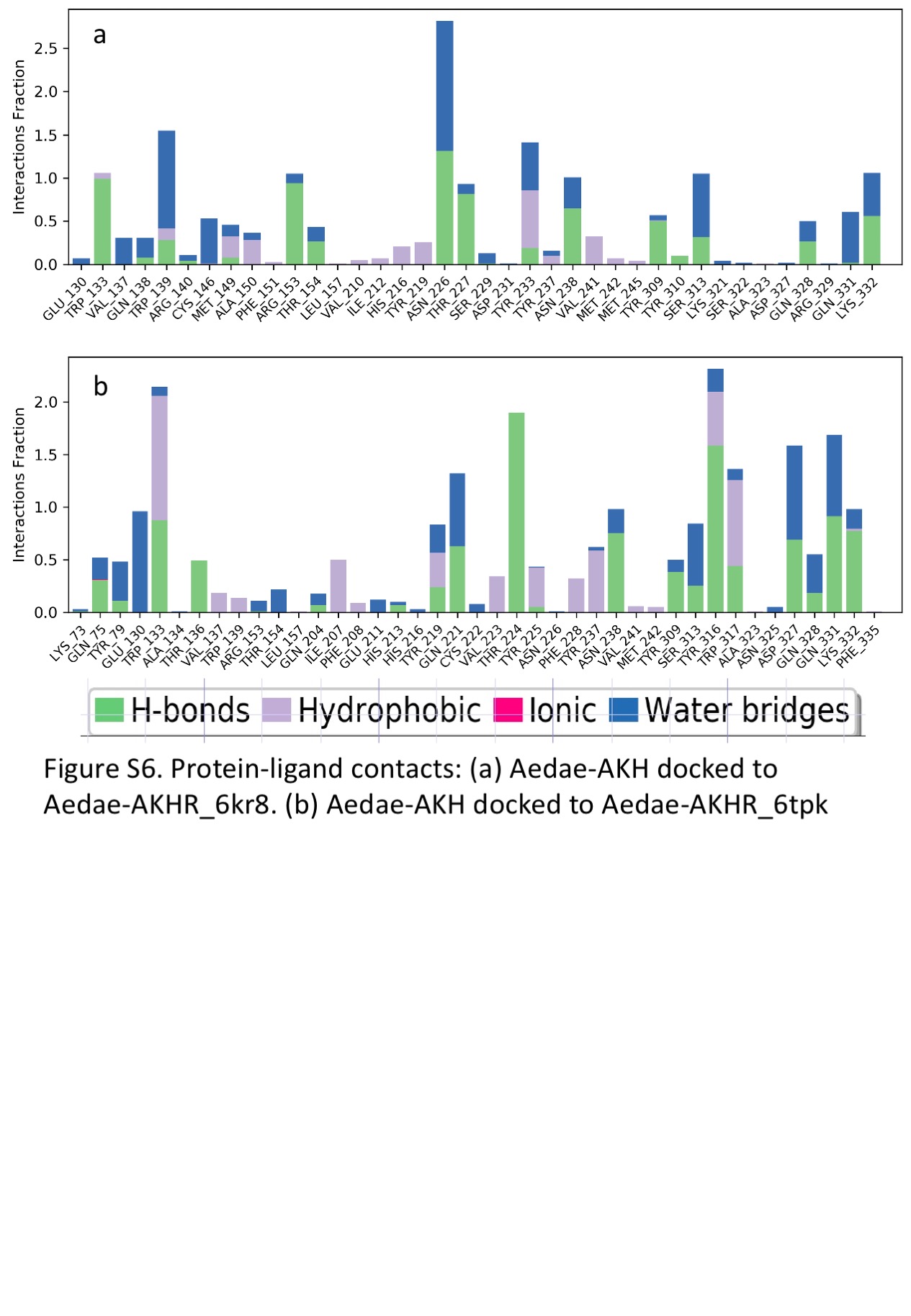

Supplement: Supplementary file 1 [file biomolecules-14-00313-s001.zip › biomolecules-2849093-supplementary/Figure S6.jpg]

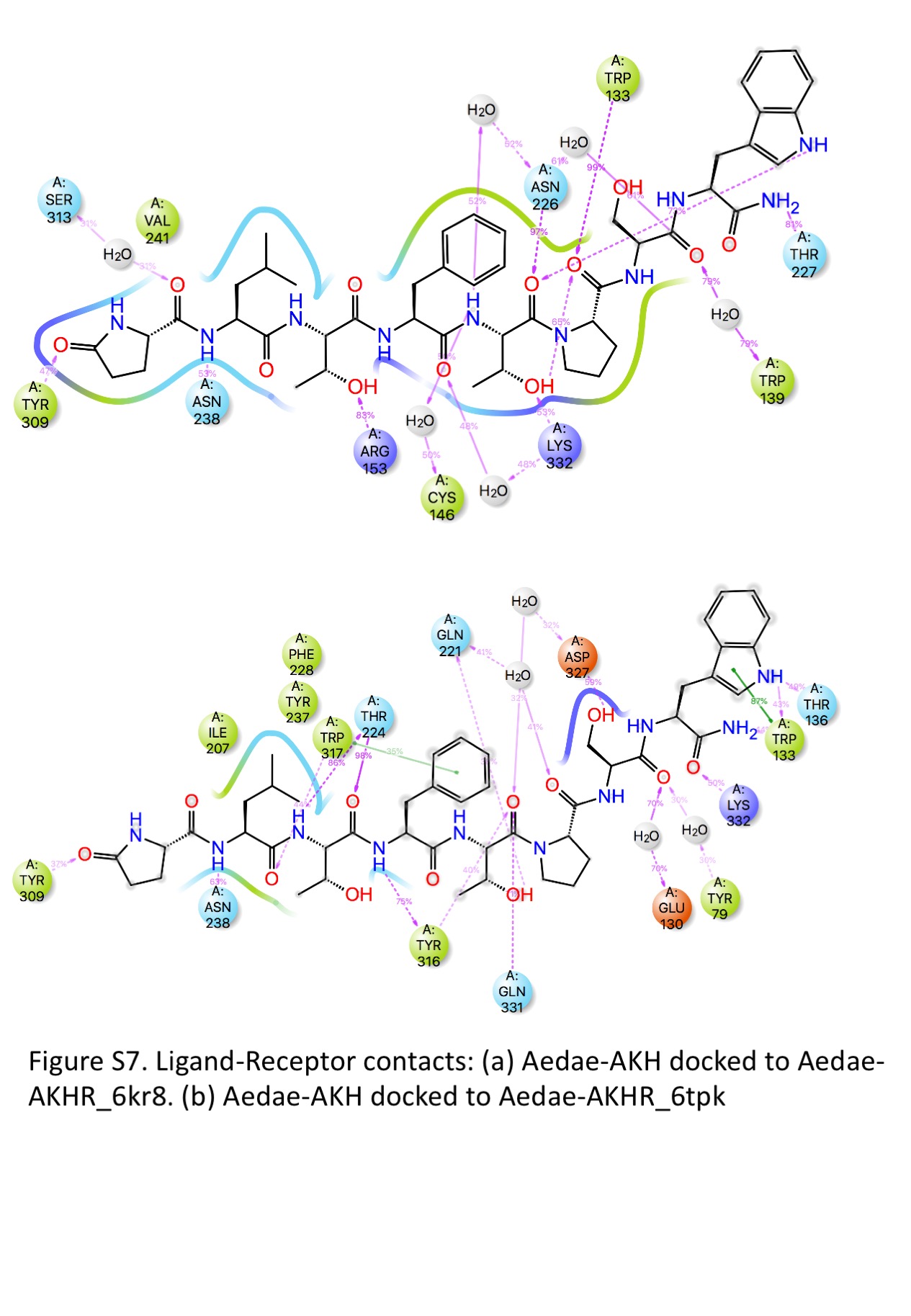

Supplement: Supplementary file 1 [file biomolecules-14-00313-s001.zip › biomolecules-2849093-supplementary/Figure S7.jpg]

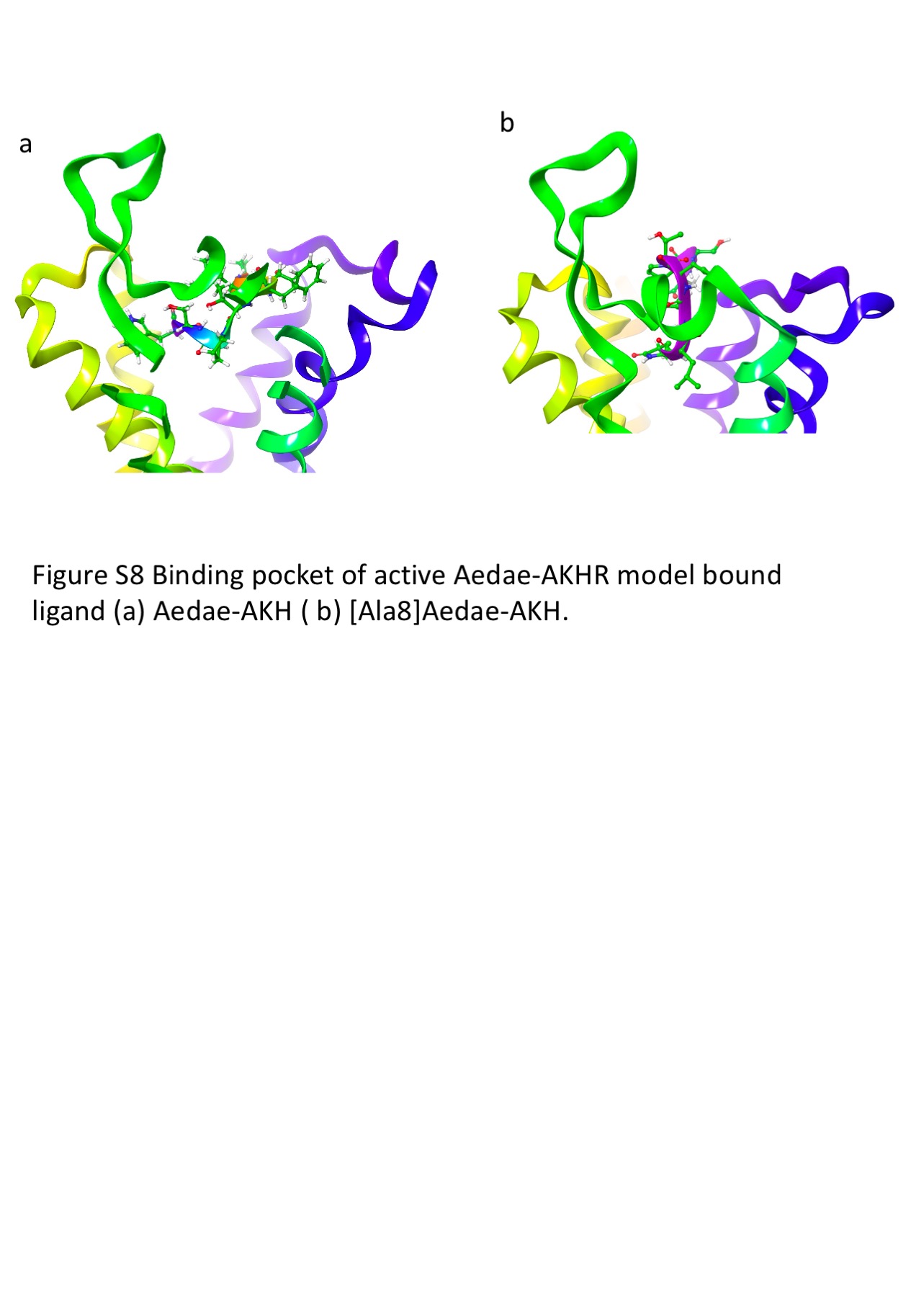

Supplement: Supplementary file 1 [file biomolecules-14-00313-s001.zip › biomolecules-2849093-supplementary/Figure S8.jpg]

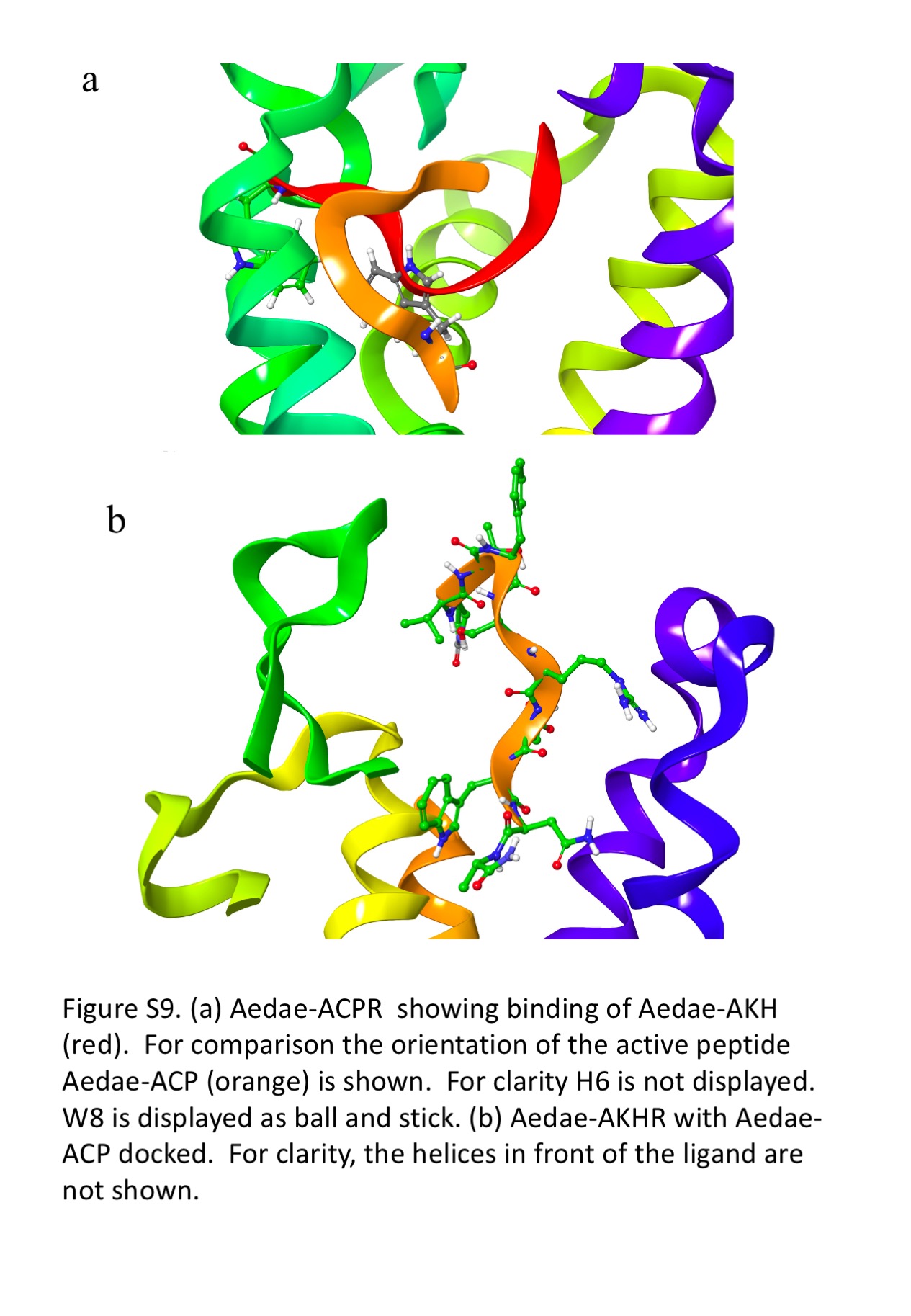

Supplement: Supplementary file 1 [file biomolecules-14-00313-s001.zip › biomolecules-2849093-supplementary/Figure S9.jpg]
